# Electrochemical synthesis of Betti bases and their application as fluorescent probes for Hg^2+^ ions

**DOI:** 10.1186/s13065-025-01638-4

**Published:** 2025-11-06

**Authors:** Eman A. El-Khouly, Amr M. Mahmoud, Hala B. El-Nassan

**Affiliations:** 1 Pharmaceutical Organic Chemistry Department, Faculty of Pharmacy, Cairo University, 33 Kasr El-Aini Street, Cairo, 11562 Egypt; 2 Analytical Chemistry Department, Faculty of Pharmacy, Cairo University, 33 Kasr El-Aini Street, Cairo, 11562 Egypt

**Keywords:** Electrochemical synthesis, Betti bases, Deep eutectic solvents, Green chemistry, Mercury ions, Fluorescent probe

## Abstract

The present study included the first electrochemical synthesis of Betti bases in deep eutectic solvents as a green and sustainable method of synthesis. The reaction conditions were optimized by varying different factors like time and type of electrode material. The prepared compounds were utilized as fluorescent probe for mercury ions. The fluorescence intensity of the synthesized Betti base derivatives **4a-e** was investigated and the highest fluorescence intensity was displayed by compound **4e** (the 2-fluorophenyl derivative). The Betti base **4e** showed excitation/emission peaks at 368/461 nm, respectively. The Betti based fluorescence was quenched by Hg^2+^ ions. The fluorescent probe responded linearly to Hg^2+^ concentration in the range of 0.2 to 10.0 µM. The limit of detection (LOD) was estimated to be 0.041 µM. The Stern-Volmer constant (K_sv_) was calculated to be 2.69 ± 0.07 × 10^5^ M^-1^.

## Introduction

Betti bases are synthesized by Mannich reaction of 2-naphthol, aryl aldehydes, and either ammonia, or amines [1]. These simple stereoactive derivatives have wide application in organic and analytical chemistry. Thus, Betti bases act as chiral catalysts in many organic reactions. Examples include their use as chiral ligands in enantioselective addition of organozinc reagents to aldehydes, and in arylation of aldehydes. In addition, they can be used to separate enantiomers and to prepare several metal ligands [1]. Betti bases were also used as efficient fluorescent sensors for the determination of the Cr^3+^ and Hg^2+^ ions in water samples [2, 3].

The wide and diverse applications of Betti bases initiated the need for more efficient and green methods of synthesis [4, 5]. These methods includes the use of catalyst [6–8] and nanocatalysts [9–12], the use of surfactants [13] and the use of microwave irradiation [8].

Deep eutectic solvents (DESs) have emerged as new and efficient green solvents and catalysts for many organic reactions in the recent years. They are considered as the green alternative of ionic liquids that can be formed more easily from readily available and environment friendly components. DESs are formed of H-bond donor and acceptor that are mixed in certain ratios mostly by heating till a permanent liquid is formed [14–20]. The synthesis of Betti bases using choline chloride: urea (1:2) was reported in 2017 by Azziz et al. [21].

Organic electrochemistry (EC) is another green method of synthesis that received considerable attention in the past two decades. EC utilizes electrons as reactants and heterogenous catalysts. This eliminates the need for hazards oxidizing and reducing agents and meanwhile prevents the large amounts of wastes generated using these reagents. Therefore, EC is an atom economic, energy efficient and green method of synthesis. Indeed, EC was used for the synthesis of many organic rings and raw materials [22–30].

The combination of EC and DES proved to be an effective synthetic strategy as reported by our lab [31–34]. The use of DES eliminated the need for supporting electrolyte, reduced the voltage needed to reach the required constant current due to the high DES inherent conductivity and shortened the reaction time needed to complete the reaction.

Various organic small-molecules based fluorescent probes have been reported in the literature for sensing of Hg^2+^ ions in water samples including: rhodamine, boron-dipyrromethene (BODIPYs), coumarin, pyrene, phthalic anhydride, indole, chromenone, 1,8-naphthalimides, lysine, phenothiazine, thiocarbonyloxadiazole, triphenylamine–triazines, tetraphenylethene, peptidyl and semicarbazone. Moreover, macrocyclic compounds have been exploited for Hg^2+^ ions detection such as crown-ether, calix [4]arene, cyclodextrin as reviewed in the literature [35–38].

These sensors can be either “off-on” fluorescent sensors where signal is enhanced in the presence of the analyte or “on-off” fluorescent sensor where the fluorescent signal is quenched in the presence of the analyte. Betti bases were previously reported as efficient “off-on” fluorescent sensors for the determination of Hg^2+^ ions concentration [2].

**Scheme 1:**
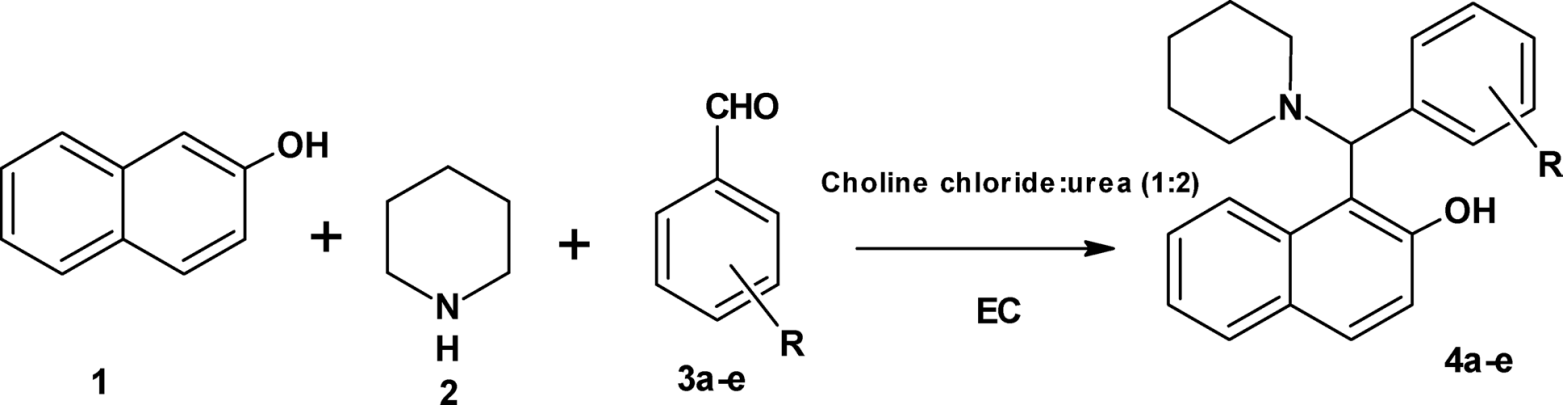
Synthesis of Betti bases **4a-e**

The present study involved the first synthesis of Betti bases under EC conditions in DESs (Scheme 1). The prepared compounds were used as “on-off” fluorescent probe for the detection of Hg^2+^ ions.

## Results and discussion

### Preparation of Betti base 4a in DESs

A preliminary study was conducted using conventional synthetic method by heating at 80 °C in a water bath to determine the most suitable DES for the synthesis of Betti base. Thus, a mixture of 2-naphthol, piperidine and 4-chlorobenzaldehyde was allowed to react in different DES (Table 1). The DESs used are formed of choline chloride and urea, thiourea, ethylene glycol, zinc chloride, oxalic acid or malonic acid. The results indicated that the reaction was successful in both choline chloride: urea and choline chloride: zinc chloride but with low yield of the product **4a** after 1 h. While, the reaction did not take place in all other DESs.

**Table 1:** Synthesis of 1-((4-chlorophenyl)(piperidin-1-yl)methyl)naphthalen-2-ol (**4a**) in DES^a^

Entry	DESs	Yield (%)^b^
1	Choline chloride /urea 1:2	38
2	Choline chloride/thiourea 1:2	NR
3	Choline chloride/ethylene glycol 1:2	NR
4	Choline chloride/ZnCl_2_ 1:2	25
5	Choline chloride/oxalic acid 1:2	NR
6	Choline chloride/malonic acid 1:2	NR

^a^ Reaction condition: **1** (2 mmol), **2** (2 mmol), **3a** (2 mmol), in solvent (5 mL), heat at 80 °C in awater bath for 1 h

^b^ Isolated yield, NR: No reaction

### Preparation of Betti base 4a under electrochemical conditions in DESs

The electrochemical synthesis of compound **4a** was conducted using choline chloride: urea (1:2) as a DES (Table 2). The use of DES eliminated the demand for additional supporting electrolyte due to the high conductivity of DES. This result was consistent with our previous work [31–34]. The reaction was conducted at constant current 20 mA at 80 °C to ensure complete solubility of the starting materials. The reaction yield was 54% after 1 h (entry 1, Table 2). Trials to increase or decrease the reaction time were accompanied by a noticeable decrease in the overall yield (entry 2,3, Table 2). Changing the electrode material resulted in significant increase in the yield especially when copper was used as cathode. The highest yield (83%, entry 6, Table 2) was obtained upon using copper as a cathode and platinum as an anode. It is noteworthy that the yield obtained under electrochemical conditions was much higher than that obtained by conventional heating in DES which provided an evidence for the catalytic role of the electrochemical method. Further explanation of this role was obtained from the cyclic voltammetry study.

**Table 2:** Synthesis 1-((4-chlorophenyl)(piperidin-1-yl)methyl)naphthalen-2-ol (**4a**) under electrochemical conditions in DES^a^

Entry	Cathode	Anode	Time (min)	Yield (%)^b^
1	Graphite	Graphite	60	54
2	Graphite	Graphite	30	33
3	Graphite	Graphite	90	46
4	Platinum	Graphite	60	NR
5	Graphite	Platinum	60	54
6	Copper	Graphite	60	62
7	Copper	Platinum	60	83

^a^ Reaction condition: **1** (2 mmol), **2** (2 mmol), **3a** (2 mmol), in Ch. Cl/urea 1:2 (5 mL), heat at 80 °C, C.C. 20 mA

^b^ Isolated yield

### Preparation of Betti bases 4b-4e under electrochemical conditions in DES

The scope of the reaction was investigated using different aldehydes **(**Table 3**).** Compounds **4b-4e** (all are halogen substituted) were obtained in low to moderate yields under the optimized EC conditions. Trials to prepare derivatives using aldehydes with electron donating groups like methyl and methoxy were unsuccessful. The structure of compounds **4a-4e** was confirmed by their ^1^HNMR spectra that showed a singlet signal at δ 5.30–5.72 ppm corresponding to the CH proton which also appeared in the ^13^CNMR spectra as a signal at δ 56.7–69.7 ppm.

**Table 3:** Synthesis of 1-((substituted phenyl)(piperidin-1-yl)methyl)naphthalen-2-ols **4a-4e** under electrochemical conditions in DES^a^

Cpd No.	Ar	Yield (%)^b^	Mp (^o^C)	References
4a	4-ClC_6_H_4_	83	164–165	[21]
4b	3-BrC_6_H_4_	44	162–164	[39]
4c	4-BrC_6_H_4_	12	166–168	[21]
4d	2-ClC_6_H_4_	30	150–152	[10]
4e	2-FC_6_H_4_	38	142–144	--

^a^ Reaction condition: **1** (2 mmol), **2** (2 mmol), **3a-e** (2 mmol), in Ch. Cl/urea 1:2 (5 mL), heat at 80 °C for 1 h at C.C. 20 mA using Cu as cathode and Pt as anode

^b^ Isolated yield

### Investigation of the reaction mechanism

**Fig. 1:**
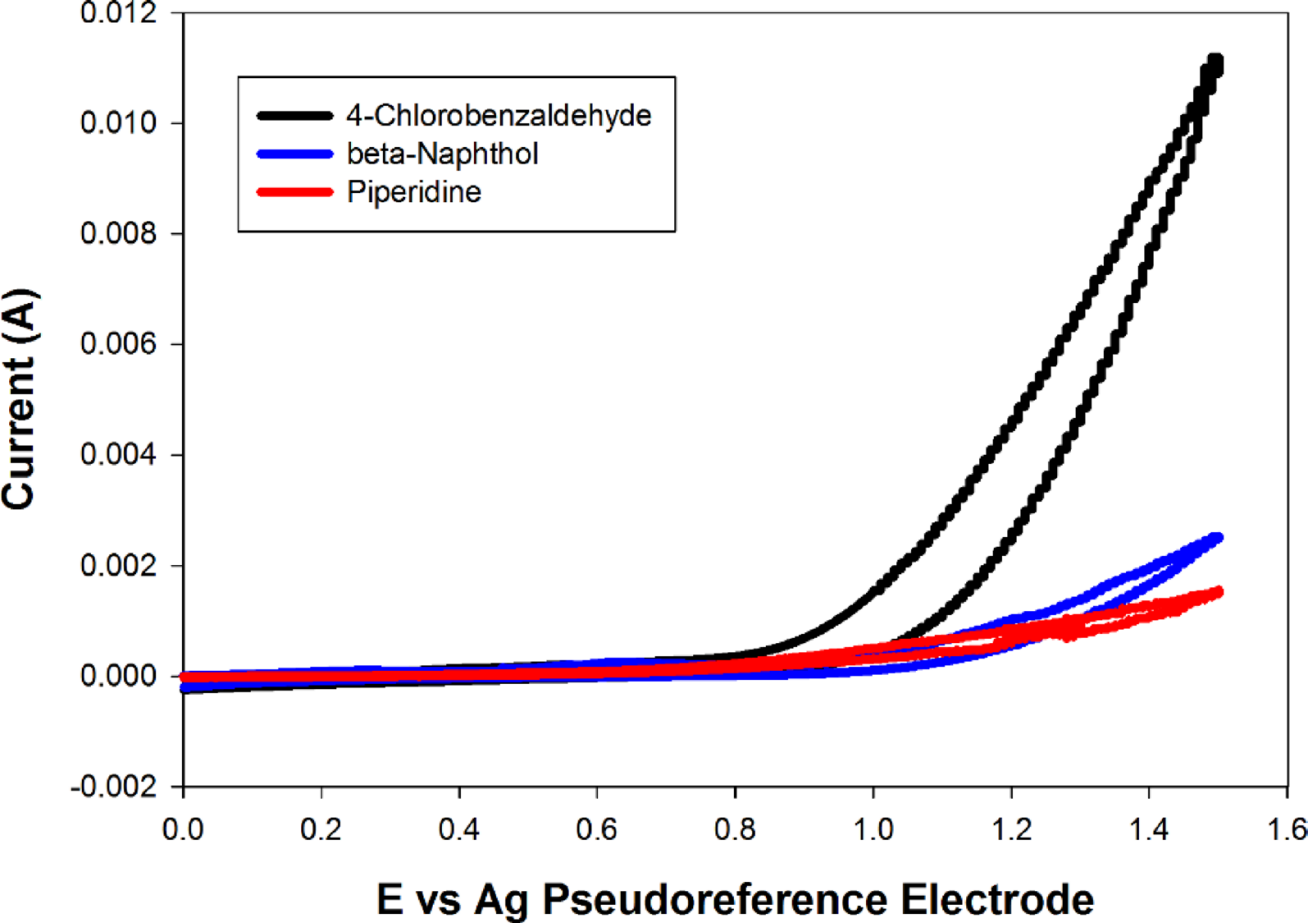
Cyclic voltammograms of 10 mM 4-chlorobenzaldehyde (black curve), 10 mM piperidine (red curve), and 10 mM 2-naphthol (blue curve) in in DES (choline chloride/ethylene glycol 1:2) at PGE surface vs. Ag/AgCl at scan rate 40 mV/sec

The reaction was traced using cyclic voltammetry to determine the reaction mechanism under electrochemical conditions. The cyclic voltammogram (Fig. 1) revealed a strong oxidation peak for the aldehyde at 0.81 V vs. Ag pseudo-reference electrode.

**Fig. 2:**
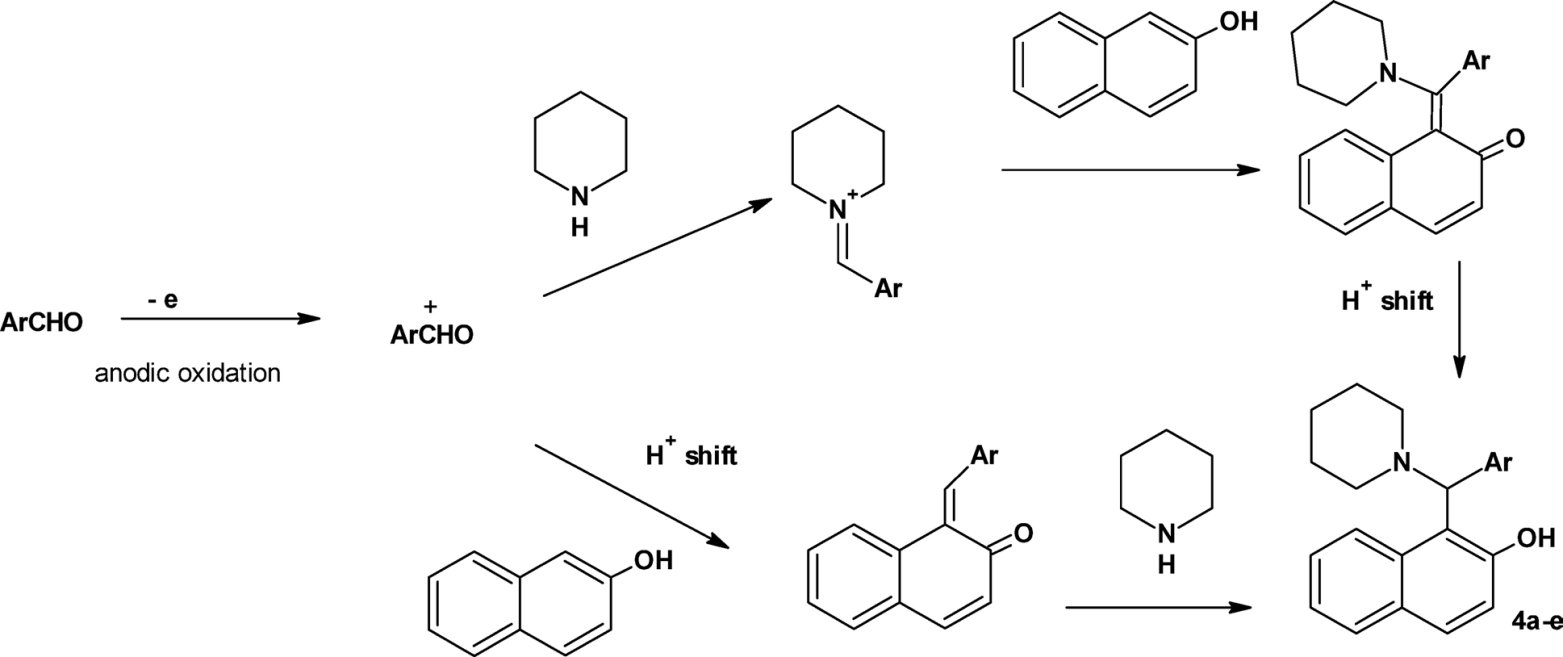
The possible pathways for the formation of Betti bases under EC conditions in DES

Thus, the mechanism of the reaction involves oxidation of the aldehyde which was then reacted with 2-naphthol to form arylmethylene derivative. Nucleophilic addition of the amine on the arylmethylene derivative afforded the target Betti bases. The DES also activated the carbonyl group of the aldehyde to enhance its nucleophilicity. Alternatively, the activated aldehyde may react with piperidine to form iminium ion. The latter react with 2-naphthol to form the target compounds (Fig. 2).

### Fluorescence study of the synthesized Betti bases

**Fig. 3:**
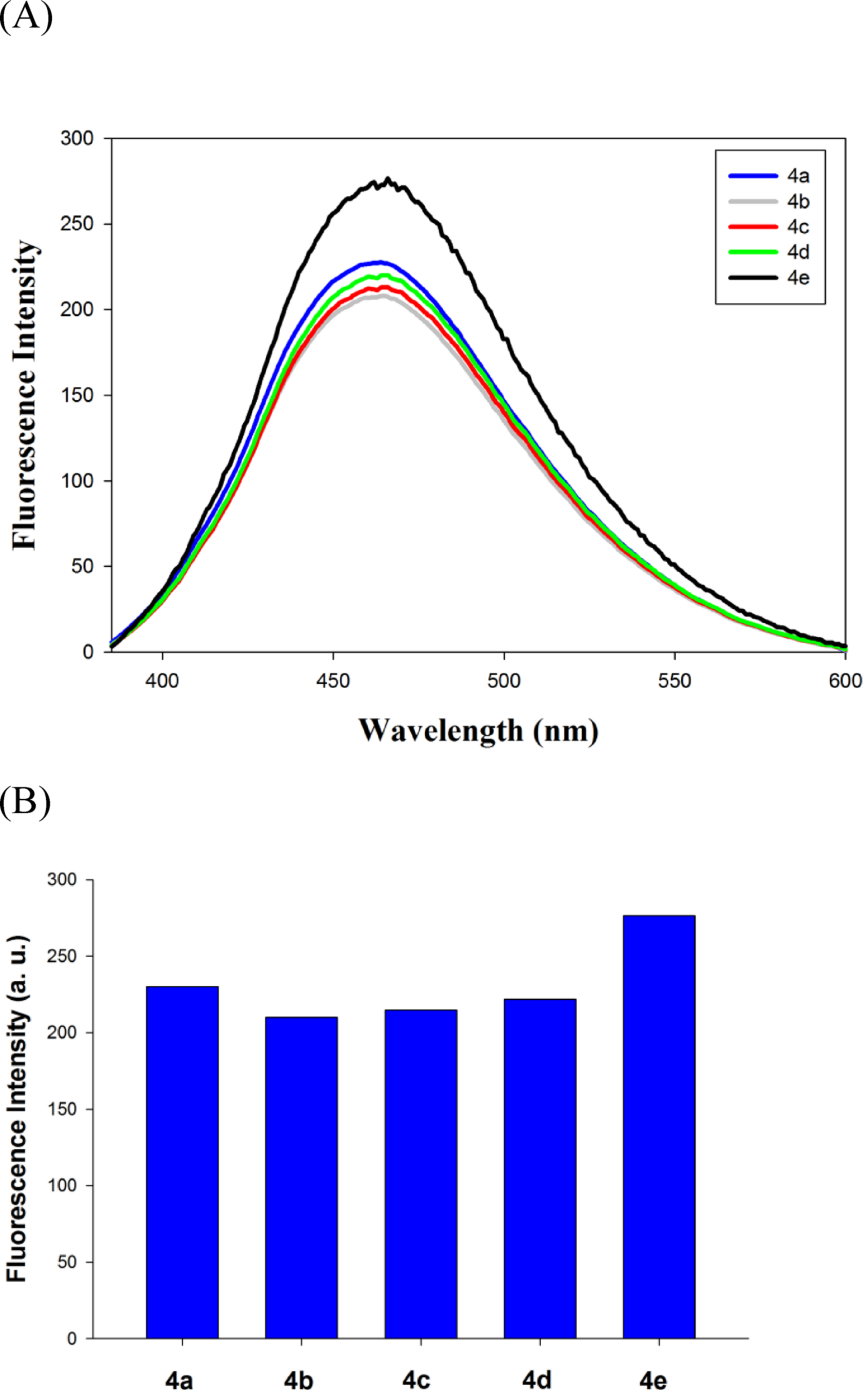
(**A**) Fluorescence emission spectra of Betti base derivatives **4a-e**. (**B**) Fluorescence intensity of Betti base derivatives **4a-e**

**Fig. 4:**
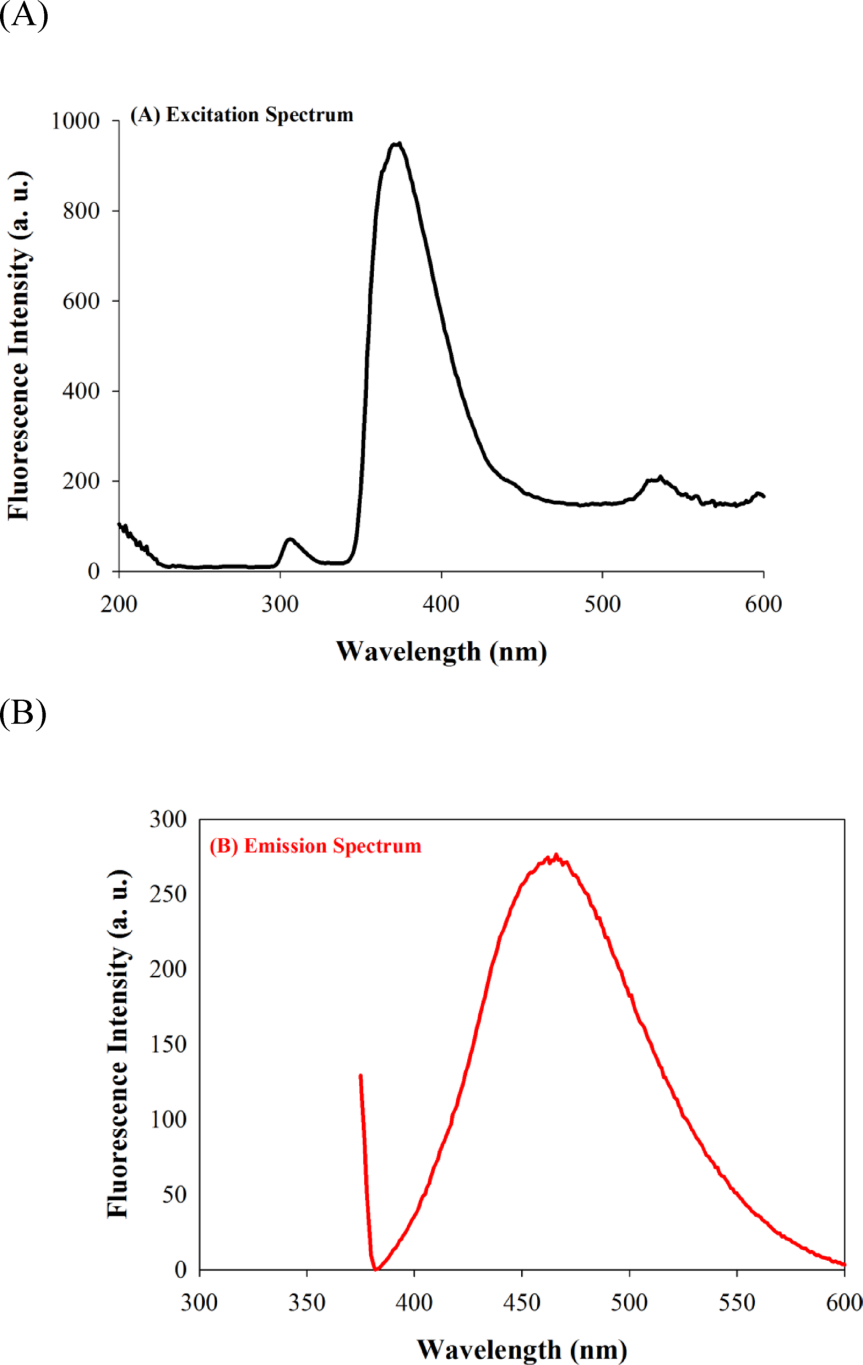
(**A**) Excitation spectrum of compound **4e**, showing excitation maximum at 368.0 nm. (**B**) Emission spectrum of of compound **4e** showing maximum at 461.0 nm

The fluorescence characteristics of the Betti base fluorescent probes was performed using Cary Eclipse fluorescence spectrophotometer. The fluorescence spectra and the intensity of the synthesized Betti base derivatives **4a-e** was investigated as presented in Fig. 3A and B, respectively, where the compound **4e** (the 2-fluorophenyl derivative) had the highest fluorescence intensity. The excitation and emission wavelengths of compound **4e** were determined by a fluorescence scan to be 368.0 nm and 461.0 nm; respectively as shown in Fig. 4A and B. The quantum yield for compound **4e** was calculated to be 0.23 following the procedure reported in our recent work [40, 41].

### UV-spectroscopic characterization of interaction between Betti base and Hg^2+^ ions

**Fig. 5:**
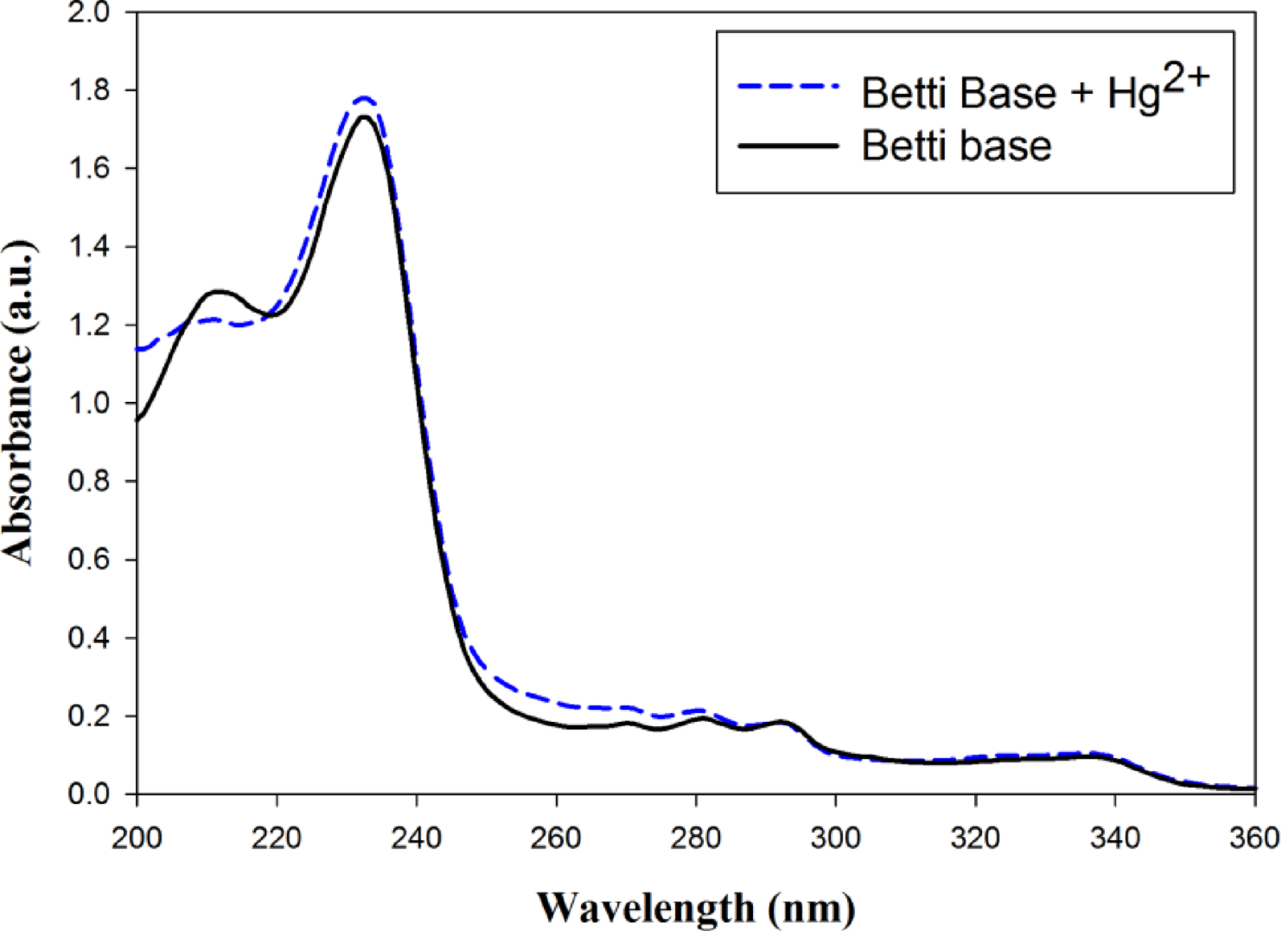
UV-spectra of Betti base compound **4e** (10 µg/mL) in DMF and Betti base compound **4e** in DMF with Hg^2+^ ions (10 µg/mL)

The UV-spectrum of Betti base compound **4e** (10 µg/mL) was presented in Fig. 5 showing λ_max_ at 233 nm, and the calculated molar absorptivity (ε) was 58101.3 M^-1^ cm^-1^. The addition of mercuric ions (10 µg/mL) to Betti base compound **4e** resulted in hyperchromic shift at wavelengths at 233 nm and 260 nm as shown in Fig. 5. These shifts indicated a complex formation between Betti base compound **4e** with Hg^2+^ ions.

**Fig. 6:**
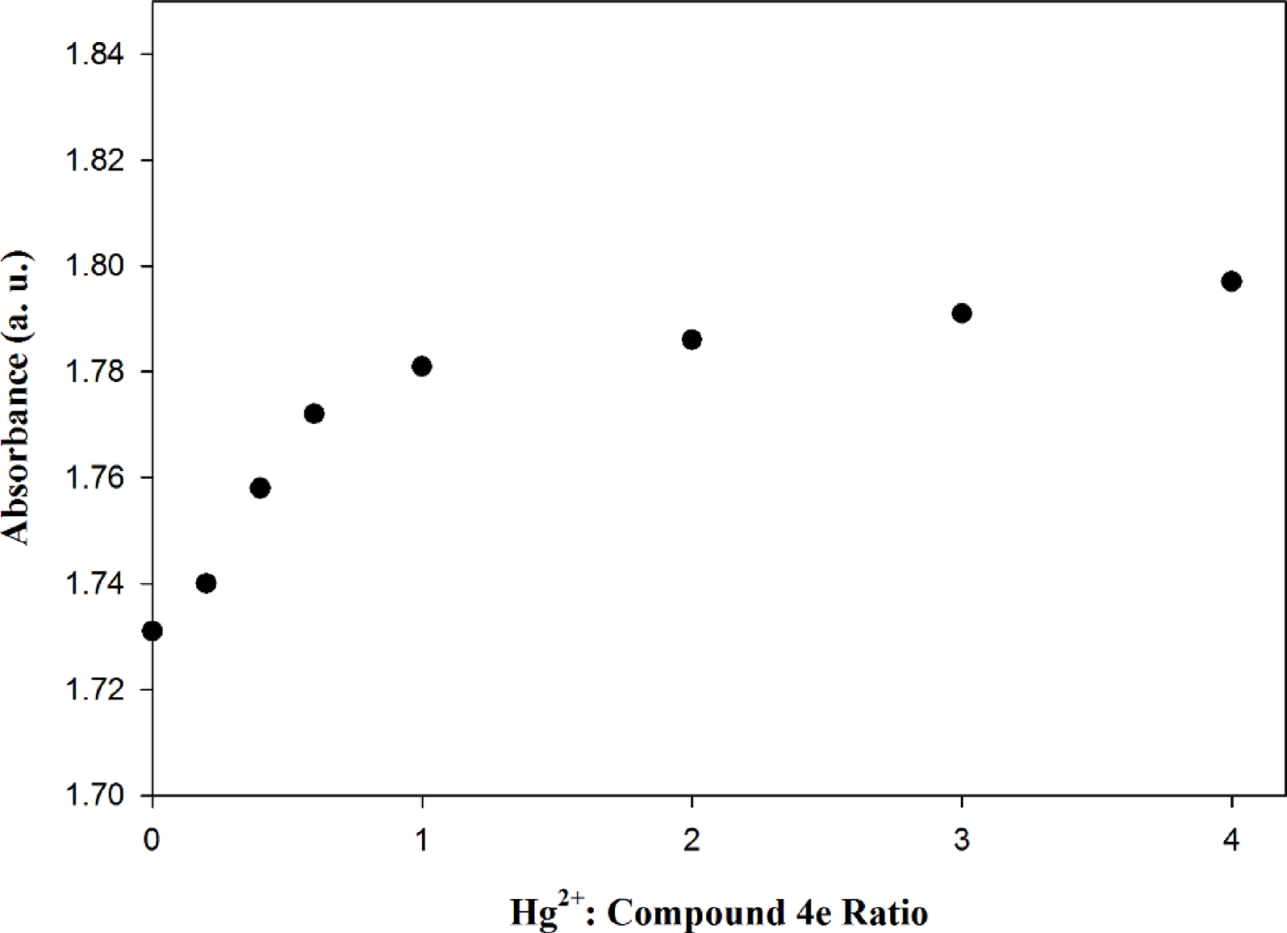
Mole ratio method for Hg^2+^ ions and Betti base compound **4e** complex

To further study the complex formed between Betti base compound **4e** with Hg^2+^ ions, the mole-ratio method has been adopted to study the stoichiometry of the complex where mixtures of solutions were prepared with a constant concentration of Betti base compound **4e** and variable Hg^2+^ concentrations and absorption was recorded at 233 nm. As shown in Fig. 6, the results suggested that the stoichiometry of the complex between Betti base compound **4e** and Hg^2+^ ions was 1:1.

### Determination of Hg^2+^ ions concentration

**Fig. 7:**
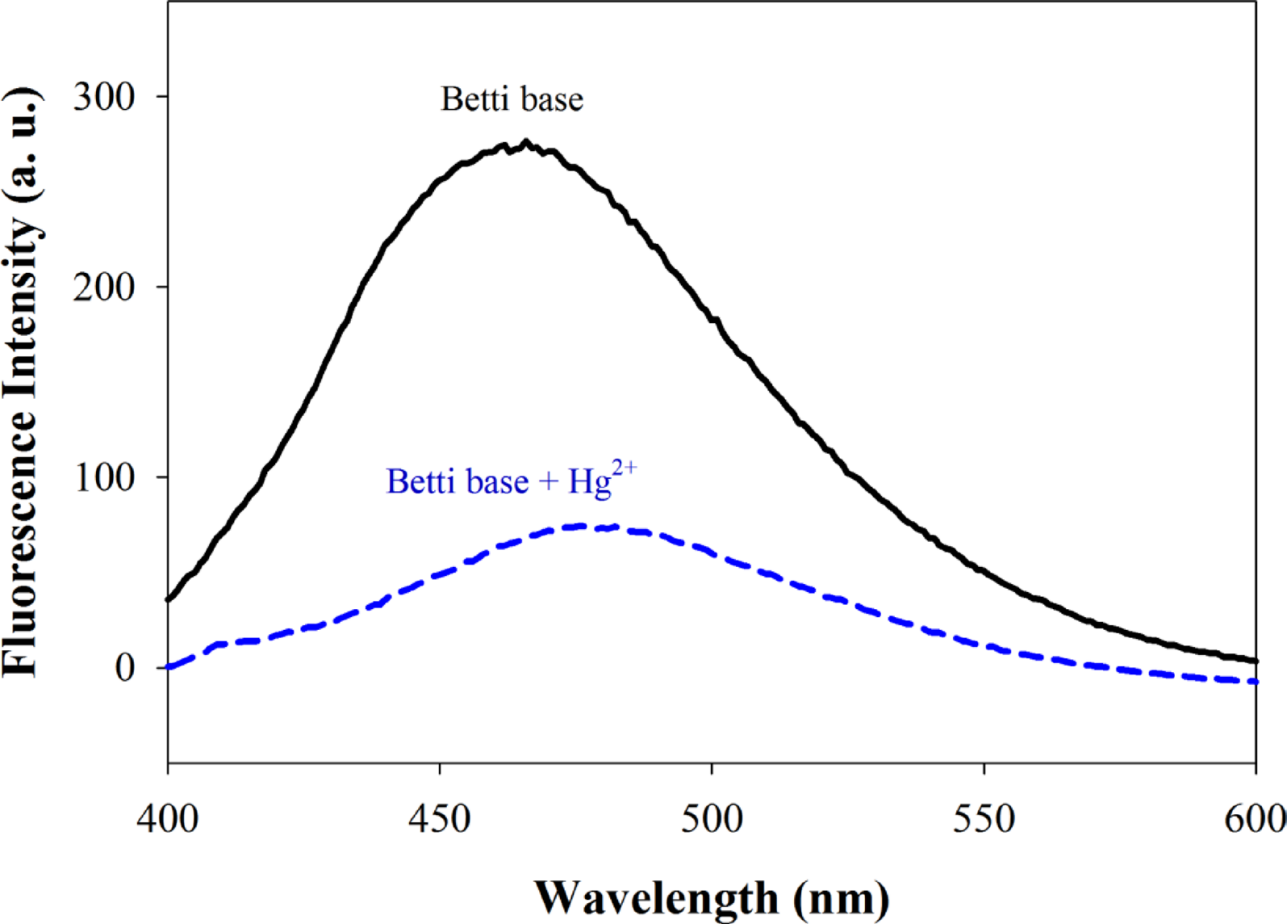
Fluorescence emission curves for Betti base **4e** alone (solid curve) and in presence of 10 µM mercuric acetate (dotted curve)

**Fig. 8:**
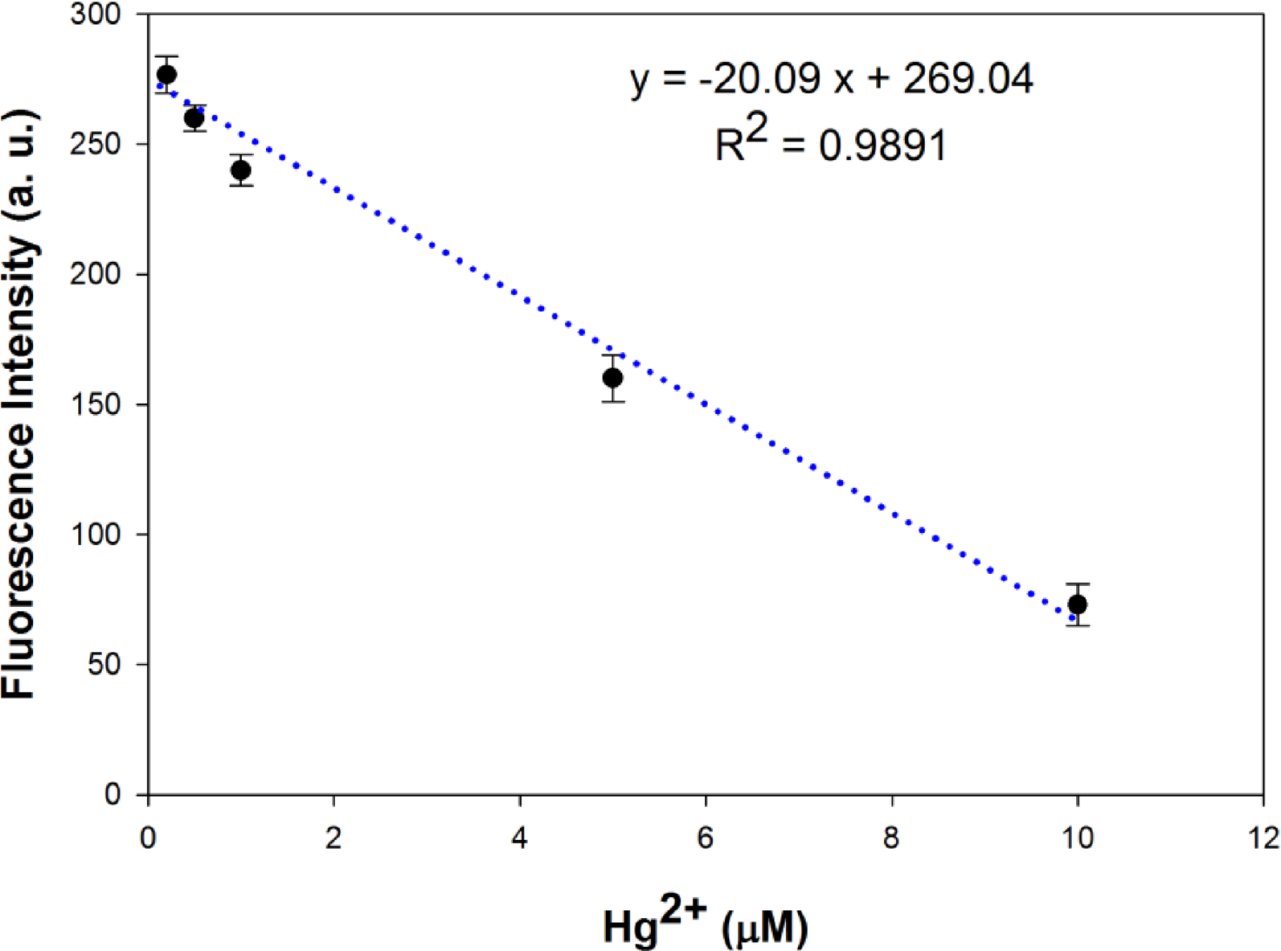
Fluorescence intensity of Betti base compound **4e** at 461.0 nm against Hg^2+^ concentrations

As shown in Fig. 7, the addition of mercuric acetate caused quenching of the fluorescence of the Betti base compound **4e**, suggesting its ability to be used as “turn off” fluorescent probe for Hg^2+^ ions detection. Plotting the fluorescence intensity at 461.0 nm of compound **4e** against the corresponding Hg^2+^ concentration allowed for the construction of a calibration curve as shown in Fig. 8. At the concentration range of 0.2–10.0 µM of mercuric acetate, linear relation was obtained with a regression equation y= −20.09 x + 269.04 and a correlation coefficient *r* = 0.9891. The limit of detection (LOD) was estimated to be 0.041 µM and the limit of quantification (LOQ) was 0.135 µM. The Stern-Volmer constant (K_sv_) was calculated to be 2.69 ± 0.07 × 10^5^ M^-1^.

**Fig. 9:**
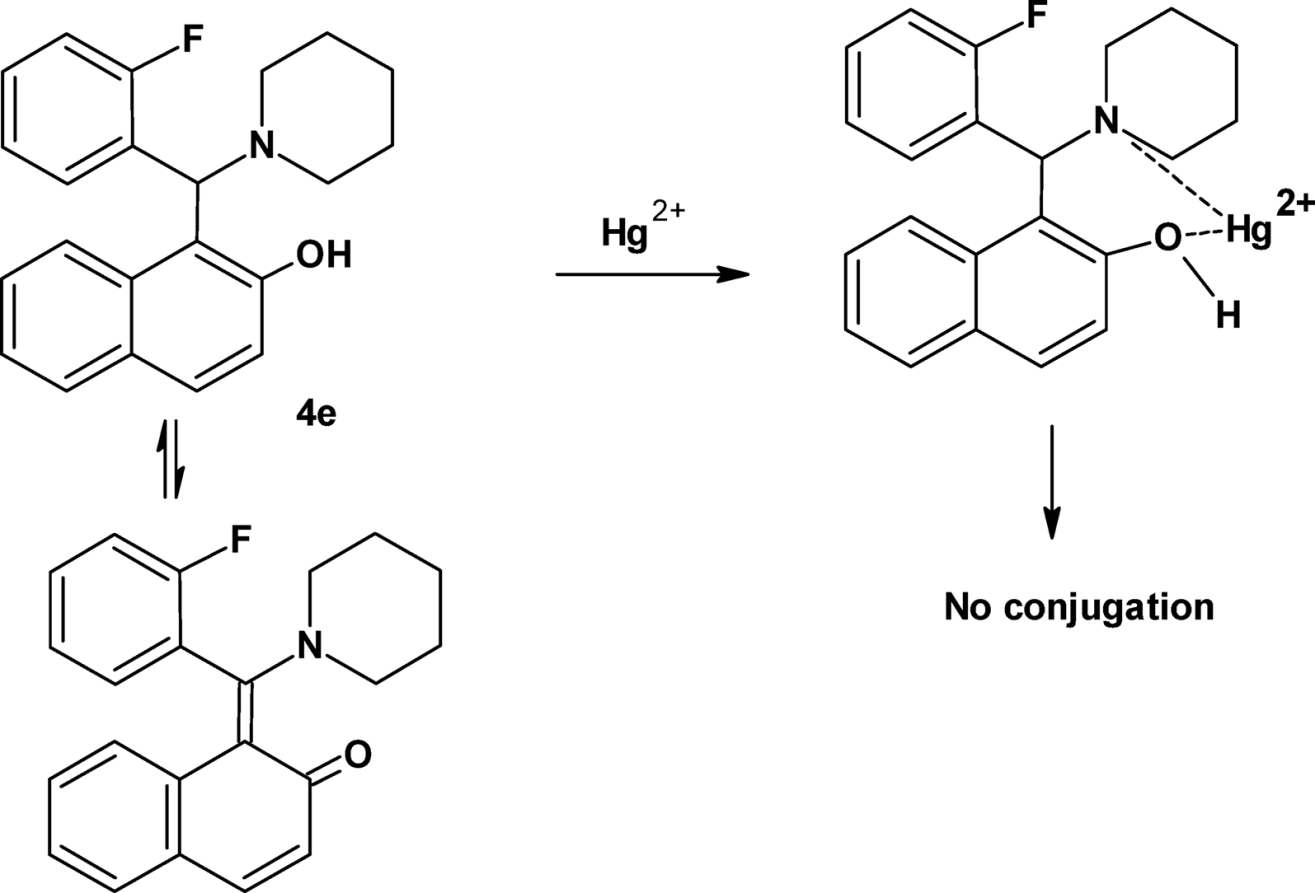
Possible interaction mechanism between betti base and Hg^2+^ ions

A possible explanation of the quenching of the fluorescence after addition of mercury ions is illustrated in Fig. 9. The addition of Hg^2+^ ions to Betti base **4e** might result in disruption of the naphthol resonance due to conjugation with the OH group.

### Selectivity study of Betti base compound 4e fluorescent probe

**Fig. 10:**
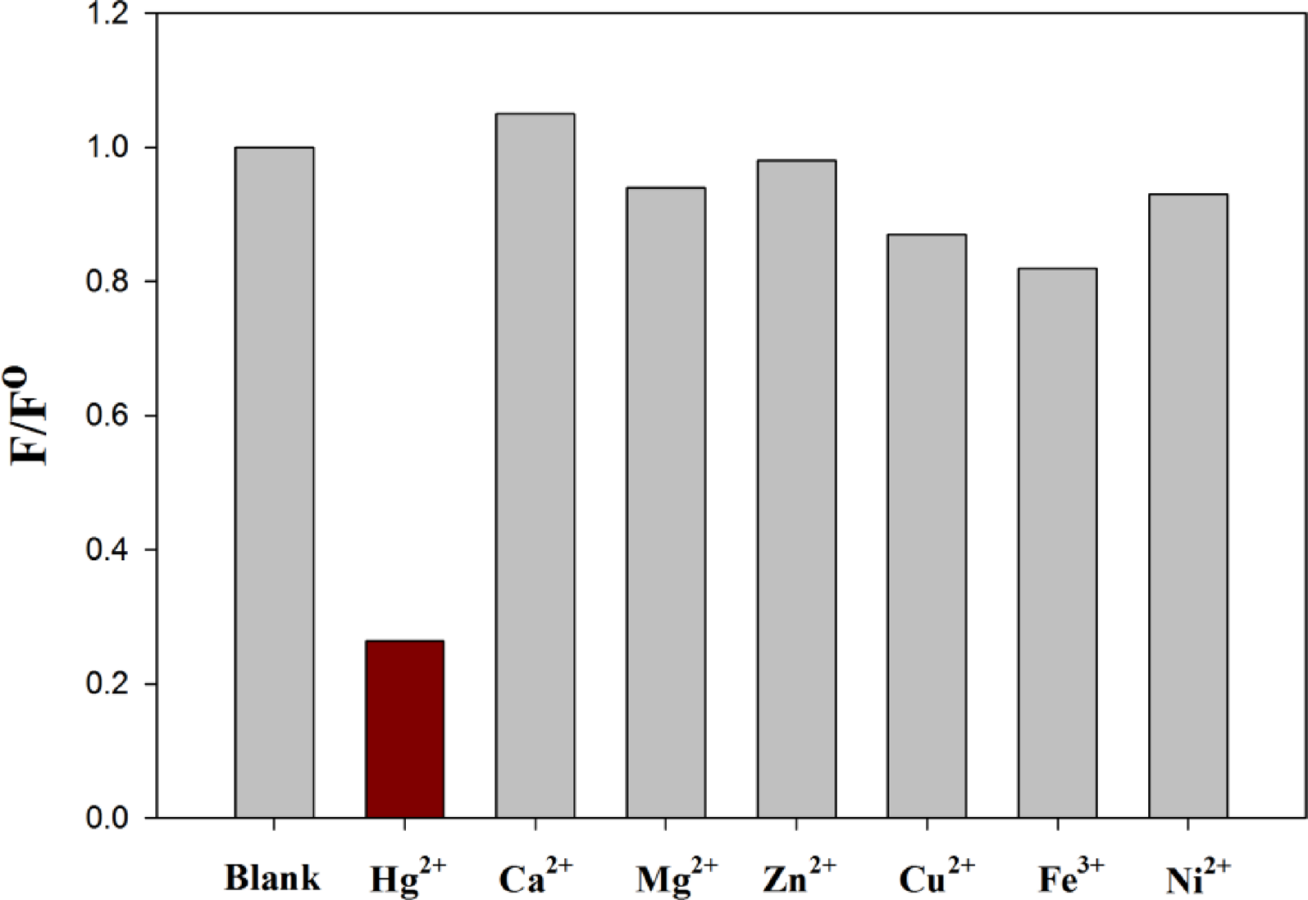
Selectivity study by comparing relative fluorescence intensity of various interfering cations with the fluorescent probe compared to control experiment

To assess the selectivity of the Betti base compound **4e** fluorescent probe towards Hg^2+^ ions, a variety of interfering cations such as Ca^2+^, Mg^2+^, Zn^2+^, Cu^2+^, Fe^3+^, and Ni^2+^ were prepared and mixed with the stock solution of synthesized Betti base compound **4e**. Figure 10 showed the relative fluorescence intensity of the interfering cations with fluorescent probe compared to fluorescent probe alone. These results indicated that Ca^2+^, Mg^2+^, Zn^2+^, and Ni^2+^ metals did not interfere with Hg^2+^ determination, while Cu^2+^ and Fe^3+^ have minor interference.

## Experimental part

### General

The melting points were determined using Stuart SMP20 apparatus. The IR spectra were recorded using Shimadzu IR 435 spectrophotometer and their values were represented in cm^-1^. The ^1^H NMR spectra, and ^13^C NMR spectra were recorded on Bruker 500 MHz and 125 MHz spectrophotometer, respectively using tetramethylsilane (TMS) as a reference compound. The Microanalyses were performed at the Regional Center for Mycology and Biotechnology, Faculty of Pharmacy, Al-Azhar University, Cairo, Egypt. The cyclic voltammetry studies were performed by Corrtest portable potentiostat/galvanostat CS100 (Wuhan, China). While, the electrochemical synthesis at constant current mode was performed using a programmable triple channel Keithley 2230-30-1 power supply (Keithley, Cleveland, Ohio, USA). Conductometric measurements were carried out with conductivity meter EcoScan CON 6 (Eutech Instruments, USA). Fluorescence experiments were performed at room temperature using Cary Eclipse fluorescence spectrophotometer (Varian, USA) controlled by Cary WinFLR program with slit width of 5.0 nm for both excitation and emission, photomultiplier tube (PMT) detector voltage had been set to medium, and a scan rate of 600 nm/min. SHIMADZU UV-Vis spectrophotometer (Model UV-1900i PC, Kyoto, Japan) controlled with UV Probe Software (Version 2.43) had been used for spectroscopic characterization of 2-fluoro derivative of Betti base (compound **4e**) in absence and presence in Hg^2+^ ions. All the chemicals and solvents were purchased from Aldrich^©^ and used without further purifications.

### Synthesis of 1-((4-chlorophenyl)(piperidin-1-yl)methyl)naphthalen-2-ol (4a) in DESs

A mixture of 2-naphthol (2 mmol), piperidine (2 mmol) and 4-chlorobenzaldehyde (2 mmol) in different DESs (5 mL) was heated in water bath at 80 °C for 1 h. The reaction mixture was poured onto water (50 mL) and the precipitate formed was filtered and recrystallized from ethanol (Table 1).

### Electrochemical synthesis of 1-((4-chlorophenyl)(piperidin-1-yl)methyl)naphthalen-2-ol (4a) in DES

A mixture of 2-naphthol (2 mmol), piperidine (2 mmol) and 4-chlorobenzaldehyde (2 mmol) was suspended in choline chloride: urea (1:2; 5 mL) in an undivided cell fitted with the appropriate electrodes (Table 2). The reaction was conducted at a constant current (20 mA) using different electrodes and times. The reaction mixture was poured onto water (50 mL) and the precipitate formed was filtered and recrystallized from ethanol.

### Electrochemical synthesis of Betti bases 4b-4e in DES

A mixture of 2-naphthol (2 mmol), piperidine (2 mmol) and the appropriate aromatic aldehyde **3b-e** (2 mmol) was suspended in choline chloride: urea (1:2; 5 mL) in an undivided cell fitted with copper as cathode and platinumt as anode. The reaction was conducted at 80 °C and constant current 20 mA for one hour. The reaction mixture was poured onto water (50 mL) and the precipitate formed was filtered and recrystallized from ethanol.

### Cyclic voltammetry study

***Apparatus.*** All cyclic voltammetric characterizations were carried out using electrochemical workstation Corrtest portable potentiostat/galvanostat CS100 (Wuhan, China). The conventional three electrodes setup was used, where the reference electrode was Ag/AgCl wire. A platinum wire was utilized as a counter electrode and the working electrode was PGE (Pencil graphitic electrode) (HB, 0.9 mm diameter).

### Preparation of standard solutions of mercuric acetate and Betti base

Stock solutions of Betti base derivatives in DMF (200.0 µg/mL) were used. Standard solutions were kept in dark place at 4˚C when not used. The mercuric acetate stock solution of 1 mM was prepared in DMF. For the measurement of fluorescence, 50 µL of compound **4e** was added in 10 mL volummetric flask and different volumes of Hg^2+^ ions were added to construct the calibration curve.

### Selectivity study of Betti base compound 4e fluorescent probe

To assess the selectivity of the Betti base compound **4e** fluorescent probe towards Hg^2+^ ions, 100 µM of a variety of interfering cations such as Ca^2+^, Mg^2+^, Zn^2+^, Cu^2+^, Fe^3+^, and Ni^2+^ were prepared in DMF, and 1 mL was mixed with the 50 µL of the stock solution of synthesized Betti base compound **4e** in 10 mL volummetric flask.

### Experimental data

#### 1-((4-Chlorophenyl)(piperidin-1-yl)methyl)naphthalen-2-ol (4a)

IR (KBr): 3360 − 3217 (OH), 2935 − 2854 (CH aliphatic) cm^− 1^; ^1^HNMR (500 MHz, DMSO-*d*_*6*_): 1.41–1.52 (m, 6 H), 2.32 (s, 4 H), 5.30 (s, 1H), 7.04–7.99 (m, 10 H, Ar-H), 13.70 (s, 1H, OH, D_2_O exchangeable); ^13^CNMR (125 MHz, DMSO-*d*_*6*_): δ 24.1, 26.0, 52.8, 69.7, 100.0, 118.0, 120.1, 121.8, 122.9, 127.0, 128.6, 129.1, 129.2, 129.7, 132.4, 132.8, 140.0, 155.5 ppm. Anal. Calcd for C_22_H_22_ClNO (351.87): C, 75.10; H, 6.30; N, 3.98; Found: C, 74.97; H, 6.42; N, 4.15.

#### 1-((3-Bromophenyl)(piperidin-1-yl)methyl)naphthalen-2-ol (4b)

IR (KBr): 3421 − 3201 (OH), 2943 − 2816 (CH aliphatic) cm^− 1^; ^1^HNMR (500 MHz, DMSO-*d*_*6*_): 1.40–1.53 (m, 6 H), 2.32 (s, 4 H), 5.31 (s, 1H), 7.05–7.08 (d, 1H, *J = 9 Hz*, Ar-H), 7.18–7.24 (m, 2 H, Ar-H), 7.36–7.37 (d, 2 H, *J = 8.5 Hz*, Ar-H), 7.60–7.61 (d, 1H, *J = 7.5 Hz*, Ar-H), 7.67–7.69 (d, 1H, *J = 8.5 Hz*, Ar-H), 7.70–7.72 (d, 1H, *J = 8 Hz*, Ar-H), 7.79-8.00 (m, 2 H, Ar-H), 13.65 (s, 1H, OH, D_2_O exchangeable); ^13^CNMR (125 MHz, DMSO-*d*_*6*_): δ 24.1, 26.8, 52.8, 69.7, 100.0, 116.4, 120.1, 121.8, 122.4, 123.0, 127.1, 128.3, 128.6, 129.2, 129.9, 131.2, 131.4, 132.4, 143.4, 155.6 ppm. Anal. Calcd for C_22_H_22_BrNO (396.33): C, 66.67; H, 5.60; N, 3.53; Found: C, 66.85; H, 5.96; N, 3.70.

#### 1-((4-Bromophenyl)(piperidin-1-yl)methyl)naphthalen-2-ol (4c)

IR (KBr): 3400 − 3300 (OH), 2935 − 2854 (CH aliphatic) cm^− 1^; ^1^HNMR (500 MHz, DMSO-*d*_*6*_): 1.42–1.54 (m, 6 H), 2.32 (s, 4 H), 5.310 (s, 1H), 7.04–7.06 (d, 1H, *J = 10 Hz*, Ar-H), 7.18–7.22 (t, 1H, *J = 10 Hz*, Ar-H), 7.33–7.37 (7, 1H, *J = 10 Hz*, Ar-H), 7.45–7.54 (m, 4 H, Ar-H), 7.66–7.69 (d, 1H, *J = 10 Hz*, Ar-H), 7.70–7.73 (d, 1H, *J = 10 Hz*, Ar-H), 7.95–7.97 (d, 1H, *J = 10 Hz*, Ar-H), 13.68 (s, 1H, OH, D_2_O exchangeable); ^13^CNMR (125 MHz, DMSO-*d*_*6*_): δ 24.1, 26.0, 52.8, 69.7, 116.0, 120.0, 121.8, 122.0, 122.9, 127.0, 128.6, 129.1, 129.7, 131.0, 132.1, 132.4, 140.1, 155.5 ppm. Anal. Calcd for C_22_H_22_BrNO (396.33): C, 66.67; H, 5.60; N, 3.53; Found: C, 66.89; H, 5.76; N, 3.68.

#### 1-((2-Chlorophenyl)(piperidin-1-yl)methyl)naphthalen-2-ol (4d)

IR (KBr): 3400 − 3240 (OH), 2962 − 2823 (CH aliphatic) cm^− 1^; ^1^HNMR (500 MHz, DMSO-*d*_*6*_): 1.53–1.55 (m, 6 H), 2.37 (s, 4 H), 5.72 (s, 1H), 7.07–7.10 (t, 1H, Ar-H), 7.19–7.24 (m, 3 H, Ar-H), 7.35–7.37 (m, 1H, Ar-H), 7.48–7.49 (m, 2 H, Ar-H), 7.52–7.71 (m, 3 H, Ar-H), 13.86 (s, 1H, OH, D_2_O exchangeable); ^13^CNMR (125 MHz, DMSO-*d*_*6*_): δ 22.2, 26.0, 59.4, 66.2, 116.1, 116.3, 118.0, 120.0, 121.0, 124.0, 124.4, 124.9, 127.3, 128.7, 129.4, 130.3, 131.2, 132.0, 148.7, 150.4 ppm. Anal. Calcd for C_22_H_22_ClNO (351.87): C, 75.10; H, 6.30; N, 3.98; Found: C, 75.34; H, 6.41; N, 4.16.

#### 1-((2-Fluorophenyl)(piperidin-1-yl)methyl)naphthalen-2-ol (4e)

IR (KBr): 3400 (OH), 2931 − 2831 (CH aliphatic) cm^− 1^; ^1^HNMR (500 MHz, DMSO-*d*_*6*_): 1.10–1.50 (m, 6 H), 2.22 (s, 4 H), 5.56 (s, 1H), 7.06–8.18 (m, 10 H, Ar-H), 13.58 (s, 1H, OH, D_2_O exchangeable); ^13^CNMR (125 MHz, DMSO-*d*_*6*_): δ 24.6, 26.1, 49.3, 56.7, 114.9, 116.2, 117.5, 117.9, 120.2, 120.8, 123.0, 123.3, 124.4, 124.8, 126.0, 127.2, 128.6, 128.7, 129.0, 129.3, 130.0, 130.6, 130.9, 132.5, 150.9, 152.8, 156.3, 161.3 ppm. Anal. Calcd for C_22_H_22_FNO (335.42): C, 78.78; H, 6.61; N, 4.18; Found: C, 78.66; H, 6.85; N, 4.29.

## Conclusion

Electrochemical synthesis in deep eutectic solvents proved to be an efficient method for the synthesis of Betti bases through Mannich reaction of 2-naphthol, piperidine and different aldehydes. Optimization of the reaction conditions were performed and the scope of the reaction indicated that aldehydes bearing halogens could be successively prepared using the optimized conditions. The Betti base **4e** showed excitation/emission peaks at 368/461 nm, respectively. The Betti bases were used efficiently as “on-off” fluorescent probe for the detection of Hg^2+^ ions. The fluorescent probe responded linearly to Hg^2+^ concentration in the range of 0.2 to 10.0 µM. The LOD and UV characterization provided an evidence for complex formation between Betti base and Hg^2+^ ions.

## Data Availability

The data supporting the conclusions of this study are available upon request from the authors.
